# 112. Ceftolozane/Tazobactam Susceptibility Trends Among Gram-negative Bacilli Collected in the United States: SMART 2016 to 2023

**DOI:** 10.1093/ofid/ofaf695.044

**Published:** 2026-01-11

**Authors:** Mark G Wise, Karri A A Bauer, John Esterly, Fakhar Siddiqui, Katherine Young, Mary Motyl, Daniel F Sahm

**Affiliations:** IHMA, Schaumburg, IL; Merck & Co, Inc, Kenilworth, New Jersey; Merck & Co., Inc., Rahway, New Jersey; Merck & Co., Inc., Rahway, New Jersey; Merck, Rahway, New Jersey; Merck, Rahway, New Jersey; IHMA, Schaumburg, IL

## Abstract

**Background:**

Ceftolozane/tazobactam (C/T) is an antipseudomonal cephalosporin combined with a β-lactamase inhibitor approved by FDA for complicated urinary tract and intraabdominal infections in adults and children, and hospital-acquired/ventilator-associated bacterial pneumonia in adults. We evaluated annual trends in the antimicrobial activity of C/T against Enterobacterales (EB) and *Pseudomonas aeruginosa* (PA) isolates collected in the United States from 2016-2023 for the global SMART surveillance program.

**Figure:**
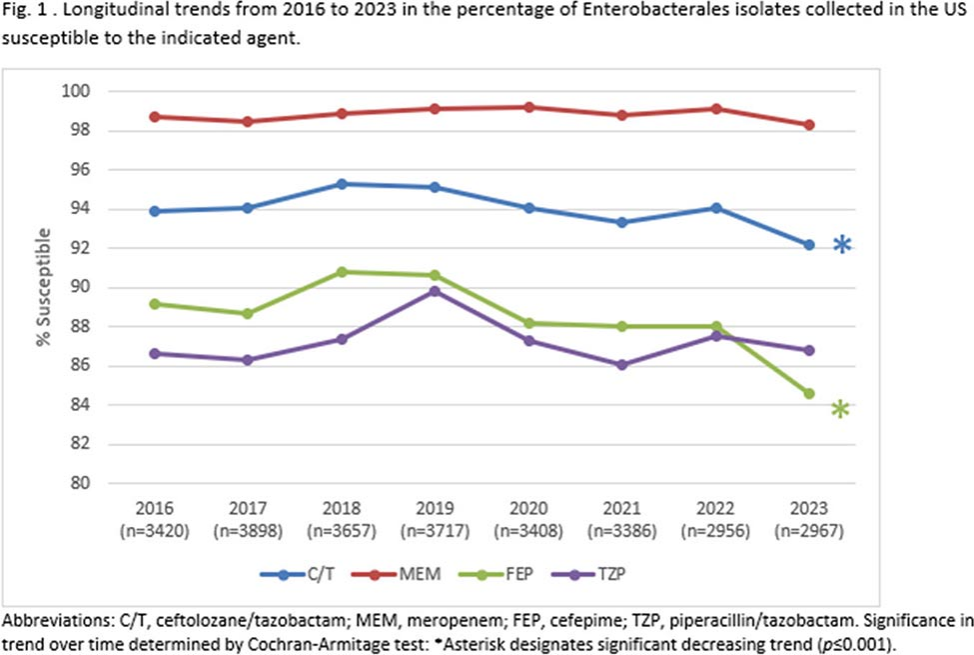

**Figure:**
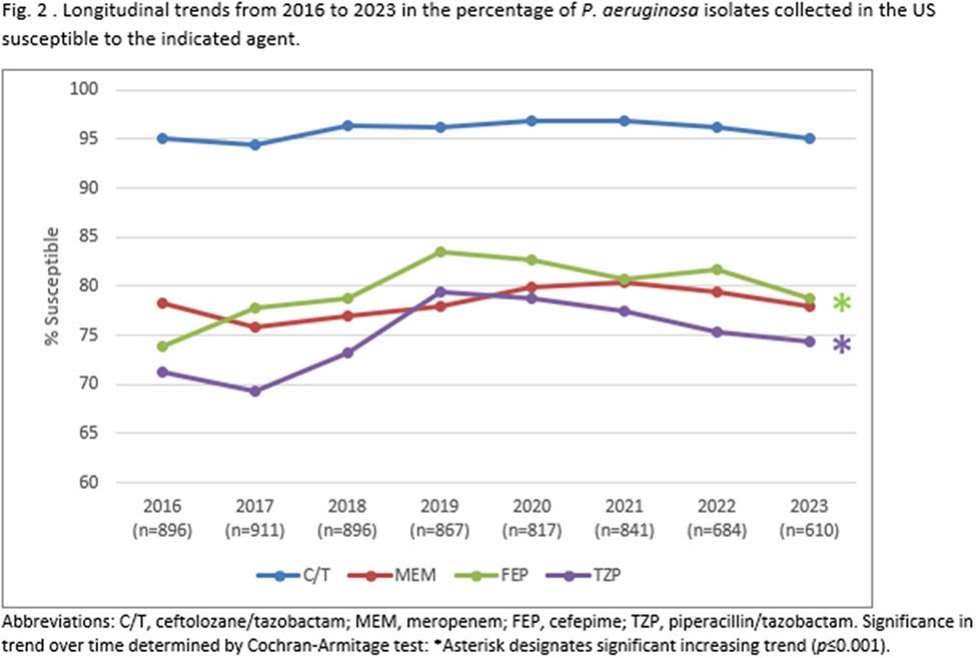

**Methods:**

From 2016-2023, 35 unique clinical laboratories in the US collected 250 consecutive, aerobic or facultative, Gram-negative pathogens per year from patients with intraabdominal, urinary tract, lower respiratory tract, and starting in 2018, bloodstream infections. Not all sites participated each year. MICs were determined using CLSI broth microdilution and interpreted with 2025 CLSI breakpoints. Trends in annual susceptibility percentages were assessed for statistical significance using the Cochran-Armitage test for trend (XLSTAT 2024.2.2.1422). A *p*-value ≤0.001 was considered significant.

**Results:**

Against EB, the annual susceptibility percentage to C/T ranged from 95.3% (2018) to 92.2% (2023) with a statistically significant decreasing trend (*p*=0.001) over the 8-year time frame (Fig. 1). Susceptibility to meropenem was consistently >98% each year, while susceptibility percentages to cefepime were approximately 5 percentage points less than C/T each year, with cefepime also exhibiting a significant decreasing trend in susceptibility (*p*< 0.0001). Against PA, C/T inhibited ≥94.5% of the population each year, approximately 16-20 percentage points higher than meropenem (Fig. 2). Susceptibility of PA to cefepime and piperacillin/tazobactam was also consistently lower than C/T, with both agents exhibiting a trend of increasing activity over the studied time range (*p*< 0.0001).

**Conclusion:**

C/T maintained consistently high levels of antimicrobial activity against PA in the United States from 2016-2023, while a trend of slightly decreasing activity levels was observed against EB. Nevertheless, C/T inhibited a considerably higher percentage of EB isolates than cefepime and piperacillin/tazobactam each year.

**Disclosures:**

All Authors: No reported disclosures

